# Association between abnormal myocardial scintigraphy findings and long-term outcomes for elderly patients 85 years or older: a retrospective cohort study

**DOI:** 10.1186/s12872-019-1240-y

**Published:** 2019-11-06

**Authors:** Takao Kato, Mitsumasa Okano, Yoshizumi Haruna, Moriaki Inoko

**Affiliations:** 1 Department of Cardiovascular Medicine, Kyoto University Graduate School of Medicine, 54 Shogoin Kawahara-cho, Sakyo-ku, Kyoto, 606-8507 Japan; 2 Cardiovascular Center, The Tazuke Kofukai Medical Research Institute, Kitano Hospital, 2-4-20 Ohgimachi, Kita-ku, Osaka, Osaka 530-8480 Japan

**Keywords:** Abnormal, Finding, Elderly, Prognosis, Scintigraphy

## Abstract

**Background:**

Normal findings of cardiac scintigraphy predict good outcomes. However, a paucity of the data exists for elderly patients 85 years or older. In the present study, we aimed to demonstrate the association between the abnormal findings of cardiac scintigraphy and the risk of all cause death in patients 85 years or older.

**Methods:**

We enrolled 143 consecutive patients 85 years or older with known or suspected coronary artery disease who underwent stress scintigraphy under adenosine or an exercise test and a ^99m^Technetium (Tc)-labeled tracer or thallium 201 (^201^Tl), dual tracer rest scintigraphy using ^201^Tl and ^123^I-β-methyl iodophenyl pentadecanoic acid (^123^I-BMIPP), or ^123^I-BMIPP single tracer scintigraphy. Ischemia was defined by an induced perfusion abnormality according to a provocation test with recovery at rest or decreased uptake of ^123^I-BMIPP despite normal perfusion at rest. Infarction was defined by perfusion abnormalities assessed by images at rest on ^201^Tl or ^99m^Tc-labeled tracer. We defined these findings as abnormal when at least one of these aforementioned characteristics was observed.

**Results:**

Patients in the abnormal findings group (*N* = 62) were more likely to have undergone prior coronary angiography and to have decreased ejection fraction than those in the normal findings group (*N* = 81). The median follow-up duration was 797 days (interquartile range, 635–1045 days), with follow-up rates of 90% at 1 year and 73% at 2 years. The 2-year mortality rate were significantly higher in the abnormal findings group than in the normal findings group (26.8% vs. 10.9%; *p* = 0.01). The risk of abnormal findings relative to normal findings remained significant for the mortality (adjusted hazard ratio, 5.99; 95% CI, 1.37–42.8; *P* = 0.015).

**Conclusion:**

Abnormal myocardial scintigraphy findings were associated with the increased risk for mortality, even for patients 85 years or older.

## Background

In developed countries, the population is aging [1]. It is estimated that the prevalence of people 65 years or older will triple from 2010 to 2050, and the oldest age group (85 years or older) will account for up to 17% of the elderly population [1]. However, in Japan, the oldest age group already reached 15% in 2017. Furthermore, due to the progress of medical therapies and revascularization techniques, the life expectancy of patients with acute coronary syndrome has improved [2]. Therefore, it is important to evaluate the ability of diagnostic testing to convey meaningful information in this group of patients. Although the significance of the normal myocardial perfusion imaging for patients 75 years or older was reported for those who were scheduled to undergo non-cardiac surgery [3, 4], there is a paucity of data for patients of more advanced age in Japan. In the present study, we aimed to evaluate the prognostic value of abnormal (or normal) scintigraphy findings for patients aged 85 years or older, which is a terminal age category of current public health [5].

## Methods

### Study population

We retrospectively analyzed 143 consecutive patients aged 85 years or older with known or suspected coronary artery disease who underwent stress scintigraphy under adenosine or an exercise test and ^99m^Technetium (Tc)-labeled tracer or thallium-201 (^201^Tl), dual tracer rest scintigraphy using ^201^Tl and ^123^I-β-methyl iodophenyl pentadecanoic acid (^123^I-BMIPP), or ^123^I-BMIPP scintigraphy with normal electrocardiogram (ECG) results, at Kitano hospital from 2010 to 2013. We investigated comorbidities, the presence of typical exertional chest pain, and prior coronary angiography results, scintigraphy findings, and outcomes from their medical records. The study protocol conformed to the ethical guidelines of the 1975 Declaration of Helsinki and was approved by the institutional review board of Kitano hospital. Informed consent was waived because this was a retrospective study. We disclosed the details of the present study to the public as an opt-out method and the notice clearly informed patients of their right to refuse enrollment. Follow-up data were collected by reviewing hospital charts, by contacting the referring physicians by mail, or by contacting the patients or relative. When contacted for follow-up visit, no patients refused to participate in the study viewing the opt-out statement. Patient records and information were anonymized prior to analysis.

### Methods of nuclear imaging

#### Myocardial perfusion study using ^99m^Tc-tetrofosmin, ^99m^Tc-sestamibi, or ^201^Tl

A myocardial perfusion study was conducted using ^99m^Tc-tetrofosmin or ^99m^Tc-sestamibi with 259–300 MBq for the stress study and 740 MBq for the rest study, or ^201^Tl with 111 MBq [6]. The stress type was symptom-limited or > 85% of the maximum heart-rate exercise using a bicycle ergometer (*n* = 8) and pharmacological administration of adenosine (*n* = 88). A dual-headed single-photon emission computed tomography (SPECT) system equipped with low-energy collimators (Cannon Medical Systems Ltd., Tochigi, Japan) was used for data acquisition with a 360-degree acquisition. ECG-Gated SPECT was performed using a division of 16-frames per cardiac cycle at least at rest. We reconstructed standard short-axis and long-axis images using Butterworth and ramp filters, without scatter correction or attenuation correction. Quantitative-gated SPECT software was used to calculate left ventricular functional parameters.

#### Metabolic imaging using ^123^I-BMIP

We used 148 MBq of ^123^I-BMIPP. Myocardial perfusion SPECT proceeded simultaneously using 111 MBq of ^201^Tl (*n* = 16). With normal ECG findings, ^123^I-BMIPP without perfusion imaging was used for diagnosis (*n* = 31) based on the discretion of attending physicians. When using dual tracers, two separate 20% windows were centered with one on the ^123^I photopeak (159 KeV). and the other on the ^201^Tl photopeak (75 KeV). We did not apply cross-talk correction between ^201^Tl and ^123^I-BMIPP.

### Definitions

Ischemia was defined by an induced perfusion abnormality according to a provocation test with recovery at rest or decreased uptake of ^123^I-BMIPP despite normal perfusion at rest or without information regarding perfusion but with normal electrocardiogram results. Infarction was defined by perfusion abnormalities assessed by images at rest on ^201^Tl or ^99m^Tc-labeled tracer. We defined findings as normal when none of these aforementioned characteristics was observed. The defects measurements were performed by experienced one radiologist and one nuclear cardiologist. If one’s judgement is different from the other’s, the final judgement has been made after discussion. Ejection fraction according to quantitative gating was obtained when available. The presence or absence of comorbidities was based on the assessment of the attending physicians. The diagnostic modalities were selected based on the discretion of attending physicians. The primary endpoints were all-cause death. Every death was placed into one of the 2 categories: 1) cardiovascular deaths, which consist of heart failure (HF), myocardial infarction, sudden death, stroke, renal failure, aortic/peripheral vascular disease, and other cardiac cause; and 2) non-cardiovascular deaths, which include malignancy, infection, respiratory failure, liver failure, renal failure, bleeding, and trauma, and undetermined causes. Sudden death was defined as unexplained death in a previously stable patient. Undetermined cause of death was defined as non-sudden death subsequent with severe illness with undetermined etiology.

### Availability of data

The datasets generated and analyzed during the current study are not publicly available due to the policy of the institutional review board of our hospital, but are available from the corresponding author on reasonable request.

### Statistical analysis

Categorical variables were expressed as numbers and percentages. Continuous variables were expressed as means ± standard deviation (SD) or medians and interquartile range (IQR). Based on their distributions, the continuous variables were compared using the Student’s t-test or the Wilcoxon rank sum test between two groups.

The cumulative incidences of clinical events were estimated using the Kaplan-Meier method, and differences were assessed using the log-rank test. Multivariable Cox proportional hazards models were used to estimate the risk of abnormal findings relative to normal findings of primary outcomes. The results were expressed as hazard ratios (HRs) and 95% confidence intervals (CIs). We selected clinically relevant risk-adjusting variables (age 90 years or older, male, the presence of chest pain, diabetes mellitus, atrial fibrillation or flutter, and ejection fraction below 60%) as the primary outcome measures. Sensitivity and specificity for all-cause mortality using myocardial scintigraphy were determined using the receiver-operating characteristic (ROC) curve method after classifying patients into four groups: grade 0 (normal findings), group 1 (ischemia), group 2 (infarction), and group 3 (ischemia plus infarction).

All statistical analyses were performed by two physicians (T.K. and M.O.) using JMP 14 (SAS Institute Inc., Cary, NC). All probability values were two-tailed, and the level of statistical significance was set at *P* < 0.05.

## Results

### Baseline clinical and imaging characteristics: normal findings group versus abnormal findings group

Patients in the abnormal findings group were less likely to be 90 years or older and more likely to have undergone coronary angiography and to have decreased ejection fraction at rest than those in the normal findings group (Table 1). Scintigraphy methods were not different between the two groups.

**Table 1:** Patient characteristics and scintigraphy findings of the normal findings group versus the abnormal findings group

	Normal findings (*N* = 62)	Abnormal findings (*N* = 81)	*P* value
Mean age, years (median and IQR)	88 [86–90]	86 [85–88]	0.029
Age 90 years or older^a^, n (%)	18 (29)	10 (12)	0.018
Male^a^, n (%)	35 (56)	57 (70)	0.11
Chest pain^a^, n (%)	17 (27)	27 (33)	0.47
Hypertension, n (%)	45 (72)	59 (72)	1.00
Dyslipidemia, n (%)	24 (38)	41 (50)	0.17
Diabetes mellitus^a^, n (%)	19 (30)	29 (35)	0.59
Current or past smoking, n (%)	13 (20)	25 (30)	0.25
Body mass index > 24, n (%)	10 (16)	14 (17)	1.00
Peripheral artery disease, n (%)	3 (4.8)	9 (11)	0.23
Dialysis, n (%)	0 (0)	3 (3.7)	0.25
Atrial fibrillation/flutter^a^, n (%)	19 (30)	28 (34)	0.72
Malignancy, n (%)	4 (6.4)	4 (4.9)	0.72
Prior coronary angiography, n (%)	13 (20)	36 (44)	< 0.0001
Subsequent coronary angiography, n (%)	4 (6.4)	15 (18.2)	< 0.0001
Scintigraphy			
Adenosine provocation, n (%)	35 (56)	53 (65)	0.183
Exercise test, n (%)	4 (6)	4 (4)	
^201^TL/^123^I-BMIPP, n (%)	10 (16)	6 (7)	
^123^I-BMIPP, n (%)	13 (20)	18 (22)	
Scintigraphy findings			
Normal, n (%)	62 (100)	0 (0)	< 0.0001
Ischemia, n (%)	0 (0)	36 (44)	
Infarction, n (%)	0 (0)	37 (45)	
Ischemia and infarction, n (%)	0 (0)	8 (9.8)	
Ejection fraction at rest (mean ± SD)	73.8 ± 15.9	58.1 ± 25.4	0.011
Ejection fraction < 60%^a^	4 (6)	23 (28)	0.0009

^*201*^*Tl* Thallium 201, ^*123*^*I-BMIPP* ^123^I-β-methyl iodophenyl pentadecanoic acid

^a^Potential risk-adjusting variables selected for Cox proportional hazard models

### Clinical outcomes: normal findings group versus abnormal findings group

**Fig. 1:**
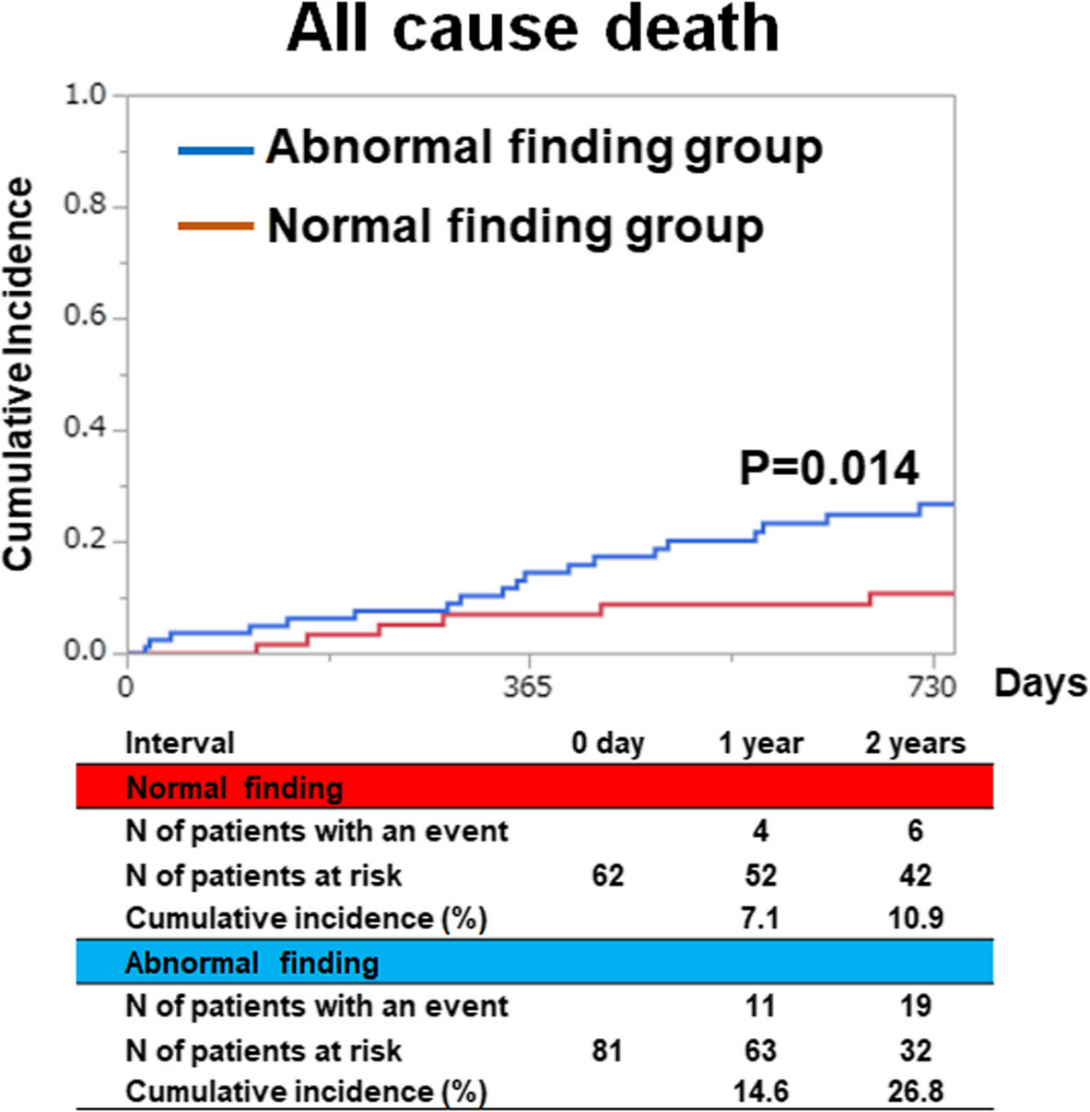
Kaplan-Meier curves of all-cause death

**Fig. 2:**
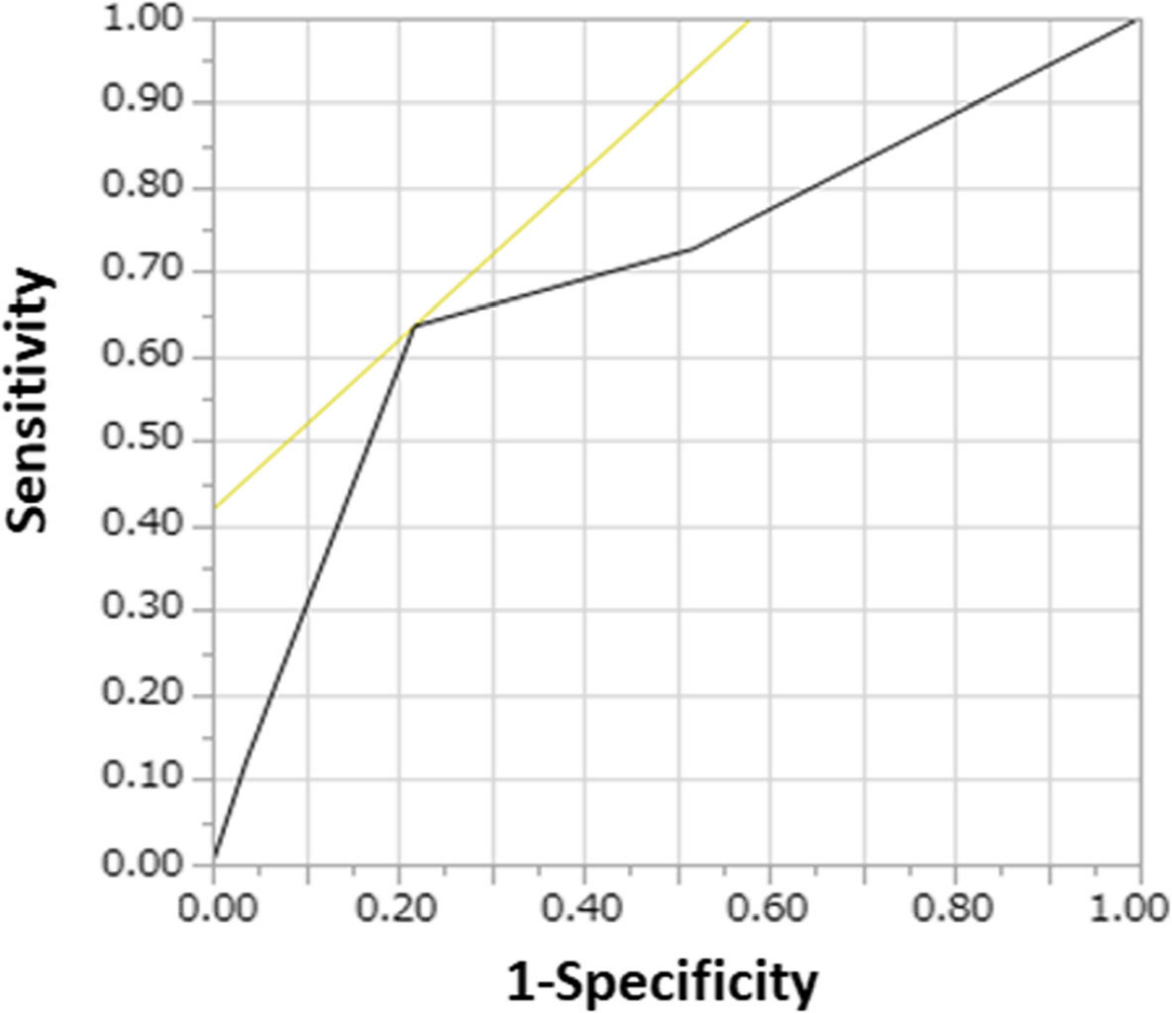
The ROC curves. Sensitivity and specificity for all-cause mortality using myocardial scintigraphy indicated that when we classified patients into four groups (grade 0: normal findings, grade 1: ischemia, grade 2: infarction, and grade 3: ischemia plus infarction), the area under the ROC curve was 0.69

The median follow-up duration was 797 days (IQR, 635–1045 days), with follow-up rates of 90% at 1 year and 73% at 2 years. The cumulative 2-year incidences of the primary endpoints were significantly lower in the normal findings group than in the abnormal findings group (10.9% vs. 26.8%; *p* = 0.01) (Fig. 1). The unadjusted risk of abnormal findings relative to normal findings was 2.46 (95%CI: 1.20–5.46: *P* = 0.013). After adjusting for confounders, the risk of abnormal findings relative to normal findings remained significant for the primary outcome measure (HR, 5.99; 95% CI, 1.37–42.8; *P* = 0.015) (Table 2). Detailed information regarding the cause of mortality is provided in Table 3. Sensitivity and specificity for all-cause mortality using myocardial scintigraphy indicated that when we classified patients into four groups (grade 0, grade 1, grade 2, and grade 3), the area under the ROC curve was 0.69 (Fig. 2).

**Table 2:** Clinical outcome

Outcomes	Normal findings N of patients with event/N of patients at risk (cumulative 2-year incidence, %)	Abnormal findings N of patients with event/N of patients at risk (cumulative 2-year incidence, %)	Unadjusted HR (95%CI)	*P* value	Adjusted HR (95%CI)	*P* value
All cause death	10/62 (10.9%)	24/81 (26.8%)	2.46 (1.20–5.46)	0.013	5.99 (1.37–42.8)	0.015

The number of patients with one event was counted through the entire follow-up period

**Table 3:** Causes of death

	Normal finding (*N* = 62)	Abnormal finding (*N* = 81)
All-cause death	9 (14.5%)	24 (29.6%)
CV death	2 (3.2%)	10 (12.3%)
	Sudden death 1, HF 1	Sudden death 3, HF 3, Vascular death 4
Non-CV death	5 (8.0%)	14 (17.2%)
	Cancer 1, Infection 4	Cancer 3, Infection 9, Renal failure 2
Undetermined	2 (3.2%)	0 (0%)

*CV* Cardiovascular, *HF* Heart failure

## Discussion

The main finding of the present study is that abnormal SPECT findings were associated with increased risk for patients 85 years or older with known or suspected coronary artery disease.

The negative predictive value of normal myocardial scintigraphy results was high in various prospective studies [7, 8]. Matsuo et al. reported that normal scintigraphy results represented a lower cardiac risk [8]. However, more than half of patients enrolled in these studies were younger than 70 years [7, 8], and patients 70 years or older were at higher risk for cardiac events compared to those younger than 70 years [8]. The novel finding in the present study is that, in the oldest patients in Japan, abnormal SPECT findings were still associated with increased risk and vice versa.

The prognostic value of SPECT has been evaluated in several studies to determine its ability to indicate abnormal findings that predict survival for elderly patients [8–12], and it was clearly reviewed by Patel et al. [13]. These studies comprised patients 70 years or older, 75 years or older, and 80 years or older, but not stratified by 85 years or older. Consistent with our data, the proportion who underwent stress imaging, especially exercise stress imaging, decreased with age [13]. Although coronary intervention has been shown to improve the survival of older patients with acute coronary syndromes [14], the trade-off between the risks and benefits was considered when making decisions regarding stable patients. Our data may provide useful information regarding the prognostic value of normal/abnormal findings of SPECT in patients aged 85 years or more, a terminal age category in public health, in Japan. This was consistent with the results of reports using positron emission tomographic imaging for patients 85 years or older in US and Europe [15]. Although further large-scale studies are required to provide stronger evidence, these findings will help practicing clinicians to make a diagnosis and treatment in the rapidly aging population in Japan.

We did not analyze the cardiovascular (CV) and non-CV death separately. Since the cause of death could not be clearly distinguished and the trends of normal and abnormal findings across the CV and non-CV death were consistent, we only analyzed the association of all cause death with scintigraphy findings. Non-cardiac causes of death are increasing in causes of death in older patients with CV disease [16–18]. An endpoint of the all cause-death may be reasonable considering the oldest patients with suspected or known CAD, as well as the those with heart failure [19]. In the present study, SPECT findings were still associated with all-cause death in oldest population, consistently with other reports in Japanese younger population [20].

There were several limitations to the present study. The main limitation of the present study was its retrospective design; therefore, selection bias may have occurred. Due to the retrospective fashion, there were no prespecified criteria for scintigraphy indications for patients with known or suspected coronary artery disease. Decisions regarding the use or non-use of diagnostic imaging and the type of diagnostic imaging used were based on the discretion of the physician. However, the physicians were experienced cardiologists who generally performed all procedures according to the guidelines for diagnosis and management of coronary artery disease. In addition, this is a single center study and there may be a classification bias about the modality selection for ischemic heart disease. Second, the tracer in the present study consisted of perfusion tracers and a metabolic tracer. Although a metabolic tracer is useful for detecting ischemia [21–23], we did not evaluate these modalities separately due to the small number of patients and events analyzed, the statistical power was limited. Third, we selected limited covariates for adjustment based on clinical relevance and avoidance of multi-collinearity. We have acknowledged that dyslipidemia, smoking, alcohol, diet, body mass index, and hypertension may have prognostic impacts [24, 25]; however, there was only solid evidence indicating that diabetes had a prognostic impact on patients 85 years or older [26]. Therefore, we included diabetes in the analysis. We did not include previous coronary angiography because it would have shown multi-collinearity with abnormal scintigraphy findings. Finally, due to the old age and limited numbers of individuals in the populations analyzed, the follow-up rate was relatively low despite the intensive effort. There might be some subjects who died in other hospitals, as a result we might overlook some of deaths.

## Conclusion

Abnormal myocardial scintigraphy findings were associated with increased risk, even for patients 85 years or older.

## Data Availability

The datasets used and analyzed during the current study are available from the corresponding author on reasonable request.

## Abbreviations

^123^I-BMIPP: ^123^I-β-methyl iodophenyl pentadecanoic acid
CI: Confidence interval
ECG: Electrocardiogram
HR: Hazard ratio
IQR: Interquartile range
SD: Standard deviation
SPECT: Single-photon emission computed tomography
Tc: Technetium
Tl: Thallium
